# Exploiting generative self-supervised learning for the assessment of biological images with lack of annotations

**DOI:** 10.1186/s12859-022-04845-1

**Published:** 2022-07-24

**Authors:** Alessio Mascolini, Dario Cardamone, Francesco Ponzio, Santa Di Cataldo, Elisa Ficarra

**Affiliations:** 1 Polytechnic University of Turin, Corso Duca Degli Abruzzi, Turin, Italy; 2 University of Turin, Via Giuseppe Verdi, Turin, Italy; 3 University of Modena e Reggio Emilia, Via Università, Modena, Italy; 4 Toscana Life Sciences Foundation, Siena, Italy

**Keywords:** Self-supervised learning, Fluorescent biological images, Generative adversarial network

## Abstract

**Motivation:**

Computer-aided analysis of biological images typically requires extensive training on large-scale annotated datasets, which is not viable in many situations. In this paper, we present Generative Adversarial Network Discriminator Learner (GAN-DL), a novel self-supervised learning paradigm based on the StyleGAN2 architecture, which we employ for self-supervised image representation learning in the case of fluorescent biological images.

**Results:**

We show that Wasserstein Generative Adversarial Networks enable high-throughput compound screening based on raw images. We demonstrate this by classifying active and inactive compounds tested for the inhibition of SARS-CoV-2 infection in two different cell models: the primary human renal cortical epithelial cells (HRCE) and the African green monkey kidney epithelial cells (VERO). In contrast to previous methods, our deep learning-based approach does not require any annotation, and can also be used to solve subtle tasks it was not specifically trained on, in a self-supervised manner. For example, it can effectively derive a dose-response curve for the tested treatments.

**Availability and implementation:**

Our code and embeddings are available at https://gitlab.com/AlesioRFM/gan-dl StyleGAN2 is available at https://github.com/NVlabs/stylegan2.

## Introduction

Thanks to their inherent capability to discover hidden data structures and extract powerful features representation, Convolutional Neural Network (CNNs) have become the fundamental building blocks in many computer vision applications. Nevertheless, much of their recent success lies in the existence of large labeled datasets: CNNs are data-hungry supervised algorithms, and thus supposed to be fed with a large amount of high quality annotated training samples [1, 2].

However, associating labels to a massive number of images to effectively train a CNN may be extremely problematic in a number of real-world applications. Significant examples are the medical and computational biology domains, where image annotation is an especially cumbersome and time-consuming task that requires solid domain expertise and, more often than not, necessitates consensus strategies to aggregate annotations from several experts to solve class variability problems [3–5]. Moreover, biological systems are affected by multiple sources of variability that make the definition of a supervised task impractical, as they require discovering new effects that were not observed during the generation of the training set. Seeking answer to such limitations, a considerable amount of literature focuses on machine learning systems, especially CNNs, able to adapt to new conditions without needing a large amount of high-cost data annotations. This effort includes advances on transfer learning [6], domain adaptation [7], semi-supervised learning [8, 9] and self-supervised representation learning [1, 10, 11]. The self-supervised representation learning (SSRL) paradigm has especially received increasing attention in the research community. Yann LeCun, invited speaker at AAAI 2020 conference [12], has defined the SSRL as “the ability of a machine to predict any parts of its input from any observed part”. In other words, SSRL can be realized by contextualizing a supervised learning task in a peculiar form (known as *pretext task*) to predict only a subset of the information and using the rest to drive the decision process. Although the pretext task guides the learning by means of a supervised loss function, the performance of the model on the pretext is irrelevant, as the actual objective of SSRL is to learn an intermediate representation capable of solving a variety of practical downstream tasks that are completely different from the pretext one. Popular SSRL pretext tasks are rotation, jigsaw, instance discrimination and autoencoder-based methods (colorization, denoising, inpainting) [1, 4]. There is a twofold explanation behind SSRL’s recent success: on one hand it can make use of the tremendous amounts of unlabeled data, heritage of the big-data era; on the other hand it is able to dispose of the human supervision and turn back to the *data’s self-supervision* [1, 4].

Current literature has primarily exploited SSRL on general category object classification tasks (e.g. ImageNet classification) [1, 4]. Surprisingly few studies have investigated how to extend SSRL methodologies to other important domains like computational biology or medicine, even though they are among the ones that are most affected by the lack of labelled training data [4]. In this regard, a longitudinal investigation by Wallace et al. [4] recently showed how traditional SSRL feature embedding fails in several biological downstream tasks. The authors suggest that the absence of canonical orientation, coupled with the textural nature of the problems, prevents classical SSRL methods from learning a pertinent representation space. They conclude that finding an optimal SSRL feature embedding for fine-grained, textural and biological domains is still an open question.

In an attempt to solve this problem, one the first works exploring image generation as a SSRL pretext task with biological images was undertaken by Goldsborough et al. [13], for the morphological profiling of human cultured cells with fluorescence microscopy. While they speculated the superiority of adversarially learned representations over autoencoder-based ones, the authors found their generative approach not competitive yet with traditional transfer learning-based methodologies [13]. More recently, a number of studies have further investigated and improved generative-based SSRL methods for biological applications, with special focus on histopathological images [14–17] and, more recently, cancer cell cultures [18]. Nonetheless, existing works typically make use of side information in the construction of their generative pretext task, that is tailored to the specific application [16].

Motivated by the the preliminary results obtained by generative-based SSRL methods with different types of microscopy images, in this study we propose *GAN Discriminator Learner* (GAN-DL), a SSRL framework that exploits the discriminator of a Generative Adversarial Network (GAN) for feature extraction, using a state-of-the-art StyleGAN2 architecture as the backbone [19]. In our framework, the adversarial training of the StyleGAN2 is exploited as a pretext task, and the trained features of the discriminator provide a new representation space to solve different downstream tasks. By doing so, GAN-DL does not make use of any annotations on the training images, nor of any side information about the specific tasks.

The idea of leveraging GAN’s discriminator as feature extractor was first introduced by Radford et al. [20], but its employment has been mainly confined to non-biological applications [21, 22]. In 2020, Mao et al. [23] showed that the effectiveness and robustness of discriminator features strongly depend on avoiding mode collapse in the network. The Wasserstein GANs family StyleGAN2 belongs to are known to be particularly resistant to the mode collapse phenomenon [22, 24]. This motivated our choice of using StyleGAN2 [19] as the backbone of our method.

**Fig. 1:**
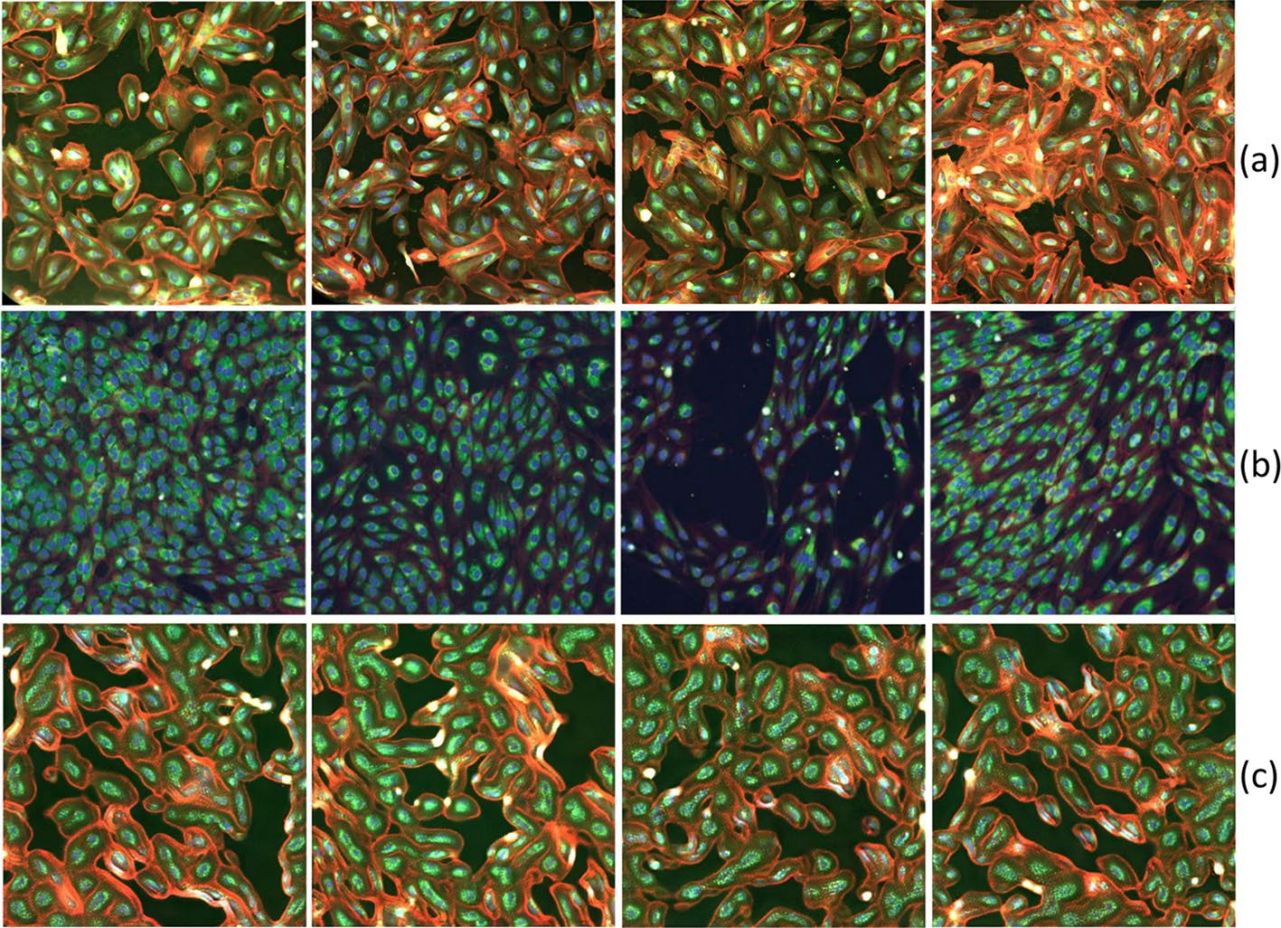
The first two rows of the figure show illustrative examples of RxRx19a [25] (**a**) and RxRx1 [26] datasets (**b**). The third row (**c**) presents representative examples of Style-GAN generated images for the RxRx19a [25] dataset

To characterize our framework, we focus on a relevant biological case-study, that is COVID-19 drug discovery, exploiting two recently released fluorescence microscopy datasets: (1) RxRx19a [25], a morphological imaging dataset that is specific of COVID-19; (2) RxRx1 [26, 27], a non-COVID related collection of fluorescent microscopy images (a more detailed description will follow). In Fig. 1 we show a representative collection of images from RxRx19a [25] (a) and RxRx1 [26] (b) datasets, depicting different cell models stained with multiple fluorescent dyes. The reported datasets perfectly embody those features (absence of canonical orientation, fine-grained content, textural nature) that make the classical SSRL pretext tasks, as described in the work by Wallace and colleagues [4], difficult, if not unsolvable.

Besides the imaging data, a transfer learning-based image embedding for the RxRx19a benchmark is also accessible online [28], which does not exploit any annotation of the target dataset. Such embedding is taken as baseline comparison to prove the goodness of our approach, and referred to as *baseline* in the rest of the manuscript.

To the best of our knowledge, the only other works addressing the problem of COVID-19 drug discovery with RxRx19a exploit labels of the target dataset in the construction of their embedding [29, 30].

**Fig. 2:**
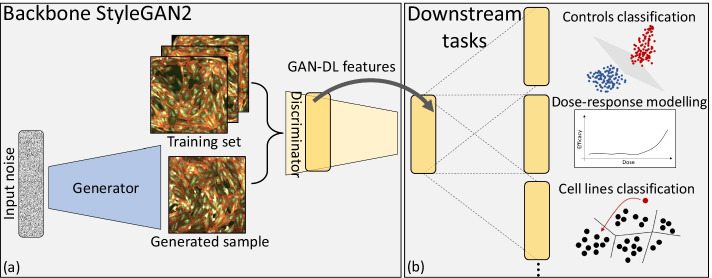
Overview of GAN-DL self-supervised representation learning framework, whose pretext task consists in the adversarial game between the generator and the discriminator of the backbone StyleGAN2 (**a**). The discriminator’s features are exploited to several downstream tasks (**b**): (1) Controls classification - classification of active and inactive compounds against SARS-CoV2 in two different cell models; (2) Dose-response modelling—disease-associated profiling from raw microscopy images; (3) Cell models classification—zero-shot representation learning classification task consisting in categorizing four different cell types

Our main contributions are the following:

1. We propose GAN-DL, a fully SSRL-based approach to characterize relevant biological case studies. We specifically employ generative SSRL in a challenging, real-world biological application of microscopy imaging tailored to COVID-19 drug discovery. We show that GAN-DL, leveraging the pretext of creating diverse and realistic images, is capable not only of proficiently managing downstream classification tasks, but also of separating multiple unrelated features at once along different axis of the latent space.
2. GAN-DL significantly deviates from the baseline featurization method proposed by Cuccarese et al. [28] and released together with the RxRx19a [25] benchmark. As a matter of fact, the authors proposed a classic transfer-learning approach featuring a deep network trained from scratch on the RxRx1 [26] and on an additional proprietary images, a very large dataset that is similar in terms of imaging technology and content to their final application, the RxRx19a [25] dataset. The necessity of a pre-training phase leveraging about 300GB of annotated microscopy images puts serious limitations to the applicability of such method in other contexts affected by scarcity of labelled data. Conversely, as above-mentioned, GAN-DL is trained solely on the unlabelled RxRx19a [25].
3. To assess GAN-DL’s ability to solve different downstream tasks, we evaluate our method on the classification of active and inactive compounds against SARS-CoV2 in two different cell lines (see Fig. 2b). We show that GAN-DL: (1) outperforms the classical transfer learning approach consisting of a CNN pre-trained on ImageNet; (2) is comparable to the baseline method in terms of accuracy, even though it was not purposely trained for the downstream tasks; (3) is able to model disease-associated profiles from raw microscopy images, without the use of any purposely labelled data during the training.
4. Finally, to assess the generalization capability of our method, we exploit the GAN-DL embedding learnt on RxRx19a in a zero-shot representation learning task consisting in categorizing the four different cell types of the RxRx1 [26] benchmark: human liver cancer cells (HEPG2), human umbilical vein endothelial cells (HUVEC), retinal pigment epithelium cells (RPE) and human bone osteosarcoma epithelial cells (U2OS).

## Materials and methods

### Dataset

The data used in this work are part of the RxRx datasets collections, that are available online [31]. More specifically, in our experiments we exploit:

1. The RxRx19a [25], which gathers several experiments aimed at investigating therapeutic potential treatments for COVID-19 from a library of FDA-approved and EMA-approved drugs or compounds in late-stage clinical trials [25]. After 24 h post-seeding, the cells have been infected with SARS-CoV-2 and then incubated for 96 h before fixation, staining and imaging. Images were produced using five channels to highlight the cell membrane and different cellular compartments, leveraging a specific fluorescent staining protocol, as described in the work by Cuccarese and colleagues [28]. The compounds were screened by treating cells in six half-log doses with six replicates per dose for each compound approximately 2 h after cell seeding. Further details about the assays protocol can be found at the official dataset website [26]. The resulting dataset is made up of 305,520 fluorescent microscopy images of size equal to $1024\times 1024\times 5$. To assess the specificity of the tested compounds, two suitable control groups have been designed. The first one consists in conditioned media preparations generated from uninfected cells (Mock), the second one is made up of cells infected in vitro by active SARS-CoV-2 virus and not treated with any compounds.
2. The RxRx1 [26], a dataset consisting of 296 GB of 16-bit fluorescent microscopy images, created under controlled conditions to provide the appropriate data for discerning biological variation in the common context of changing experimental conditions. The RxRx1 [26] has been specifically created to push innovative machine learning and deep learning pipeline on large biological datasets, aimed at drug discovery and development [26].

We leverage the whole RxRx19a [25] to train our GAN-DL on the pretext task of creating diverse and realistic images. Notably, such task does not require any specific annotation. Experiments on downstream tasks were conducted by using 75% of the control images for training and 25% for testing (randomly split by well), with all images outside of the control group used for dose-response evaluation. Images from the same wells were put in the same partition and class imbalances were corrected by automatically adjusting weights inversely proportional to class frequencies in the input data.

No images outside of the traning subset of the control group were used in the training of the downstream tasks. For both the RxRx19a and the RxRx1 we performed standard post-processing of the embedded images as described in [28], including normalization to remove inter-plate variance.

### GAN-DL’s backbone: the StyleGAN2 model

The recent literature about GANs is focused on methodologies to improve their training and counteract the well known difficulties and limitations of this phase [32]. More specifically, Wasserstein Generative Adversarial Networks (W-GANs) [24] have been introduced to prevent two common problems of training GANs. First, mode collapse is a form of GAN failure in which the network learns to generate only a subset of the data, eventually a single image or a discrete set of images representing the modes the distribution has collapsed to. The discriminator ends up trapped into a local minimum and the generator easily presents the same examples over and over to convince the discriminator. This results in a model that is heavily over-fitted on this particular subset. Second, lack of convergence due to either the generator or the discriminator, which are improving at a faster pace than the other network. This prevents the mutual improvement that is necessary for convergence.

W-GANs have proved to be an efficient solution to overcome both those limitation at once, by replacing the classical discriminator model with a critic that scores the realness of a given image by means of the so-called Wasserstein distance [24]. For our GAN-DL we employed the Nvidia’s StyleGAN2 architecture [19], that is an instance of W-GAN with residual connections in both the generator and the discriminator.

The original StyleGAN2 model has been scaled down to allow training on more reasonable hardware and time-frames. To reduce the number of parameters, we simplified the fully connected mapping network to be 3 layers deep instead of the original 8. The latent space we employ corresponds to the style vector, the sizing of which is 512 in accordance with the original paper. The network analyses each sample as a 5-channel image, with each channel containing 1 stain. To do so, the only adaptation needed over the original StyleGAN2 model is to increase the filter size of the convolutional layer closest to the image of both the generator and the discriminator to 5. Refer to Additional file 1 for the experimental setup.

### Counterpart embeddings

In our experiments, GAN-DL embedding is compared against several different counterparts:

- The RxRx19a [25] embedding released by Cuccarese et al. together with the imaging data and referred to as *baseline* in this manuscript [28]. It consists of 1024-dimensional vectors (one vector per image) obtained using a DenseNet CNN architecture specifically pre-trained for identifying the different 1,108 genetic perturbations across the four human cell types gathered in the RxRx1 dataset [25, 26]. Such dataset, which collects 125,514 high-resolution fluorescence microscopy images with corresponding labels, is a source annotated dataset with very similar imaging characteristics to the target one (the RxRx19a [25]). The author adapted their DenseNet-based network by firstly changing the initial convolutional layer to accept image input of size $512\times 512\times 5$. Like the original DenseNet model, they used Global Average Pooling to contract the final feature maps to a vector of length 2208. Then, instead of following immediately with a classification layer, the authors added a fully connected layer of dimension 1024 used as the embedding of the image. The weights of the network were learned by adding two separate classification layers to the embedding layer, one using softmax activation and the other using ArcFace activation [33], which were simultaneously optimized by training the network to recognize perturbations in the public dataset RxRx1 [25] and in a proprietary dataset of immune stimuli in various cell types, unfortunately not released by the authors. Due to operational constraints, a modified assay protocol, lacking one image channel, was used for the live-virus experiments of the RxRx19a dataset [25]. To accommodate this change, the network was trained on only the five first input channels of the RxRx1 images [25]. The proprietary model is not publicly released by the authors.
- The embedding of a DenseNet CNN pre-trained on a source dataset with completely different imaging characteristics and contents (ImageNet). For a fair comparison, the backbone of this methodology is a DenseNet, same as for the baseline solution. Pre-training a neural network with ImageNet data involves interpreting images in terms of RGB channels, while cellular images acquired by a fluorescent staining procedures, as for the generation of RxRx19a [25] and RxRx1 [26] datasets, are potentially represented by a variable number of channels. The staining procedures adopted for the RxRx datasets collection produced images of 5 channels (RxRx19a [25]) and 6 channels (RxRx1 [26]). To account for this difference, we adopted two different strategies:
  - The *ImageNet-collapsed* strategy, where we introduce a trainable convolutional layer with a kernel size of 1 at the beginning of the RGB pre-trained networks, so that the fluorescent images are converted to 3 channels pseudo-RGB images where each channel is replicated three times. The weights of such input trainable layer were learnt via fine-tuning on the given downstream task, leveraging Adam optimizer with learning rate equal to 0.001. The training lasted only for a few epochs, since the number of trainable weights is low. We picked as final model the one giving best accuracy value during training. The ImageNet-collapsed strategy features the same dimensionality as the embeddings of Cuccarese et al. [28] since it is based on the same architecture.
  - The *ImageNet-concatenated* strategy, where each channel is processed independently and then all the resulting features are concatenated. This strategy does not require any fine-tuning and produces an embedding of size 5120 ($1024\times 5$).
- The embedding of a convolutional autoencoder (referred to as *ConvAE*) trained on the target dataset RxRx19a. For this purpose, we implemented the method presented by Wallace et al. [4], that was demonstrated to be superior in term of classification accuracy to jigsaw, rotation and instance discrimination based self-supervised methods on biological images. To allow the autoencoder to converge on the higher resolution images we are evaluating, we modify the original architecture by adding the same residual connection scheme used in the generator of StyleGAN2 and GAN-DL and a perceptual loss function obtained using an Imagenet pretrained ResNet50 [34]. Refer to Additional file 1 for the ConvAE’s experimental setup. The embedding, extracted from the last layer of the encoder, features a size of 1024, same as the baseline and ImageNet-collapsed pretrained method.

Note that the embedding size varies across the different counterparts. This is constrained by the specific architecture the given featurization strategy leverages. In our GAN-DL, as mentioned in the previous subsection, the latent space we employ corresponds to the style vector, which has a size of 512.

## Results

Our experiments specifically seek an answer to two research questions: (1) is it possible to learn an accurate and reliable image featurization, able to encode and describe biological relevant information, leveraging a self-supervised pretext task?; (2) up to which extent the learned biological information can be transferred to a different dataset? To answer such questions, we have put into effect the properties of GAN-DL featurization in the following experiments.

### Visualizing GAN-DL’s representation learning capability

To characterize the representation capability of the proposed SSRL featurization methodology, we evaluate GAN-DL on the RxRx19a [25] dataset. We summarize the screening control samples into two sets of conditions, $C^+$ and $C^-$. $C^+$ represents uninfected samples treated with culture medium or a solvent, and $C^-$ represents samples infected with wild-type SARS-CoV-2 virus. For simplicity and with abuse of notation, we refer to $C^+$ as *positive controls* and to $C^-$ as *negative controls*.

In the RxRx19a [25] compound screening setting, only the images that correspond to positive and negative sets of conditions can be associated with either *live* or *dead* labels, where those labels refer to the viability of the cellular model imaged in that specific condition. The cellular model viability is unknown for the remaining part of the samples. In this regard the vast majority of the dataset is unlabelled. The large amount of unlabelled data, coupled with the textural and fine-grained aspect of the images, makes RxRx19a [25] a challenging case-study and a perfect candidate to assess our proposed SSRL methodology.

As Fig. 2 suggests, GAN-DL embedding consists of a 512-dimensional features vector. To assess and interpret its inherent capability of learning a genuine representation, we need to define a projection space able to allow some degrees of visualization of the data structure. Hence, using the control samples, we promote the explainability of the projection procedure by defining:

1. The *effectiveness-space* ${{\textbf {E}}}^2$, a two-dimensional space that represents the treatment effectiveness of the tested compounds on two axes. The *On-perturbation* axis captures the difference between uninfected samples $C^+$ and infected samples $C^-$. Intuitively, the screened compounds able to inhibit SARS-CoV-2 infection should have an *On-perturbation* value similar to the $C^+$ set of conditions. The *Off-perturbation* axis represents the remaining variability in the samples that cannot be unambiguously associated to the compound effectiveness.
2. The *cell models-space* ${{\textbf {C}}}^2$, a two-dimensional space that captures the morphological properties of the two cell models into two dimensions. The *On-perturbation* axis projects the differences of the two cell models onto one direction. Intuitively, all the samples in which VERO cells were used should have a similar *On-perturbation* value. The same goes for the samples in which HRCE cells were used. The *Off-perturbation* axis represents the remaining variability that cannot be associated to the cell model differences.

Projecting the data along *On-perturbation* and *Off-perturbation* allows us to visually represent the high-dimensional image embedding obtained by GAN-DL into two-dimensional plots. To obtain such directions, we leverage a linear Support Vector Machine (SVM) trained to classify $C^+$ versus $C^-$ (${{\textbf {E}}}^2$ space) or HRCE versus VERO control cells (${{\textbf {C}}}^2$ space). In both the cases, the separation hyper-plane fitted by the SVM and its normal respectively represent the *Off-perturbation* and the *On-perturbation* axis. As shown later in this section, the scalar projection of the 512 GAN-DL features on such spaces are exploited on one hand to provide an effective visual representation of the high-dimensional data structure through point cloud scatter plots, on the other hand to derive dose-response curves for the tested compounds. For better readability, the *On-perturbation* axis is scaled so that $C^+$ are centered around $+1$ and $C^-$ around $-1$ and the *Off-perturbation* axis is zero-centered.

**Fig. 3:**
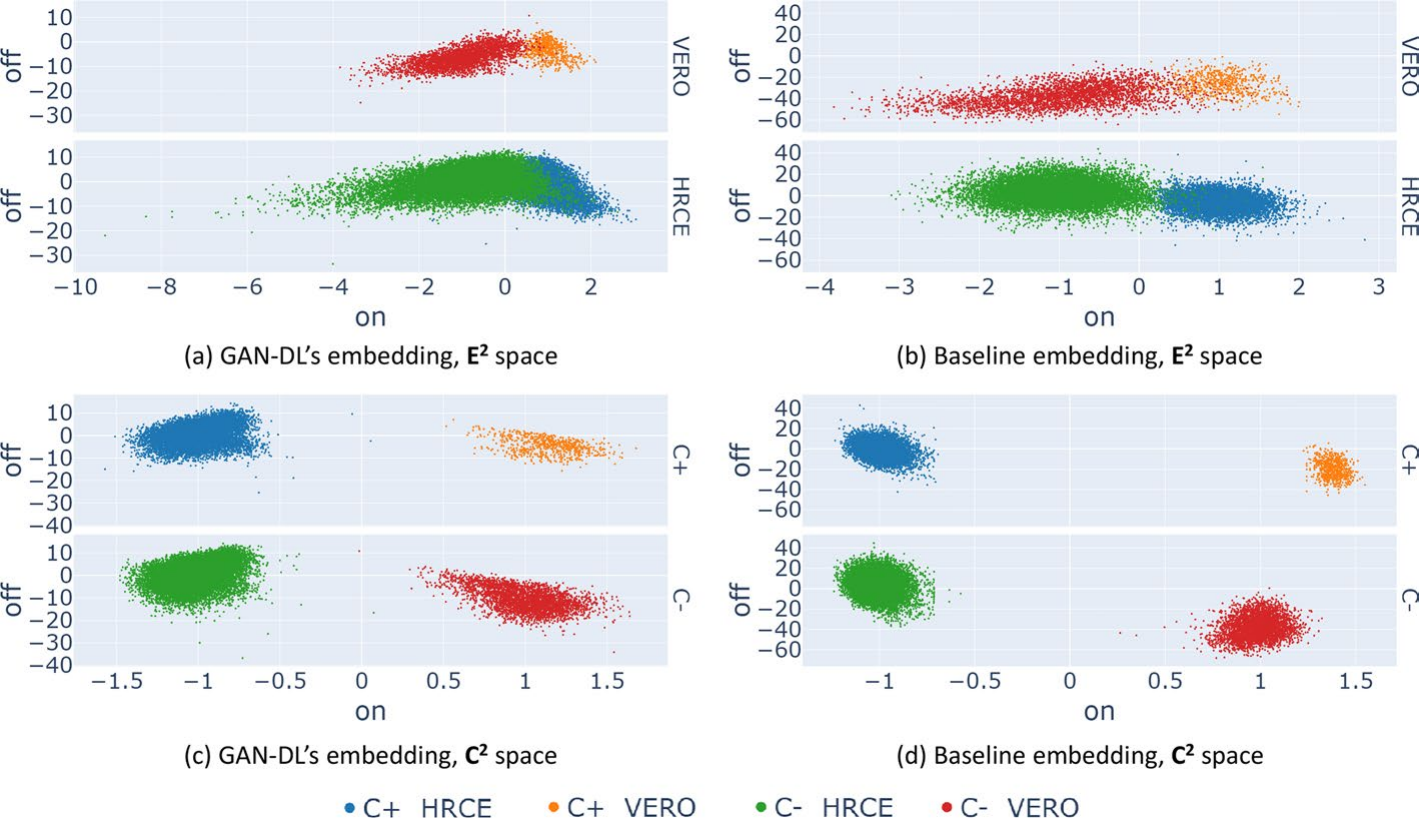
The left column of the figure shows the scatter plots of GAN-DL’s embedding of the RxRx19a [25] dataset projected onto the $E^2$ (**a**) and $C^2$ (**c**) axes. The right column shows the baseline embeddings of the RxRx19a [25] dataset projected onto the $E^2$ (**b**) and $C^2$ (**d**) axes

The plots in the first row of Fig. 3 compare our GAN-DL’s embedding (a) with the baseline embedding [28] (b) in the ${{\textbf {E}}}^2$ projection space, where we expect a degree of separation between $C^+$ and $C^-$, since such space was spanned by the SVM trained on the embeddings of the negative and positive controls. The analysis is performed considering the two sets of conditions grouped by cell model. Hence, different colors identify $C^+$ and $C^-$ for the two distinct cell models: blue and orange for the $C^+$ of HRCE and VERO cell model, respectively, green and red for the corresponding $C^-$ conditions. As it can be gathered from the degree of separation between $C^+$ and $C^-$ on the ${{\textbf {E}}}^2$ projection space, both the embeddings behave coherently in separating mock-treated samples from those where the virus was active. A quantitative comparison in terms of degree of separation between $C^+$ and $C^-$ is presented in the following subsection.

The second row of Fig. 3 shows GAN-DL featurization (c) and the baseline featurization (d) projected onto the ${{\textbf {C}}}^2$ space, where we expect a certain degree of separation between distinct cell types, irrespective of whether $C^+$ or $C^-$ are considered. Same as in the previous experiment, results are reported separately for the two cell models. Here HRCE are represented with blue ($C^+$) and green ($C^-$) colors, while VERO with orange ($C^+$) and red ($C^-$), respectively. Even in this case, the plots demonstrate that GAN-DL is able to caught the inherent variability of the two cell models, in a comparable way to the transfer-learning baseline.

### Assessing the linear separability of the controls

To assess the goodness of our embedding, we try to demonstrate that it is able to establish a good linear separability of samples on two different downstream tasks it was not specifically trained for: (1) the categorization of $C^+$ versus $C^-$ and (2) the classification of HRCE and VERO cells.

For both the tasks, the linear separability is verified by exploiting soft margin linear SVMs for classification. More specifically, we compare the classification accuracy of a linear SVM built on top of our embedding with the ones obtained by the other counterpart embeddings: (1) the baseline [28], (2) a DenseNet CNN model pre-trained on ImageNet, respectively with *collapsed* and *concatenated* strategy, (3) a convolutional autoencoder [4] trained on RxRx19a. All results presented are obtained using data that was not included in the SVM training process.

The first two lines of Table 1 report the classification accuracy values of the two classification tasks (for the first one, $C^+$ vs $C^-$, the two cellular models are merged into the same dataset). From the reported values we can observe that GAN-DL provides informative features for both $C^+$ versus $C^-$ categorization (91.4% accuracy) and cell models recognition (100% accuracy). The baseline, that leverages the RxRx1 [26] dataset as transfer learning source domain, outperforms GAN-DL by just 5% in terms of $C^+$ versus $C^-$ classification accuracy, and is equivalently 100% accurate in the other task. This is a remarkable result for GAN-DL, given that its embedding is trained on a completely different pre-text task, which does not require any kind of image annotation. Lastly, GAN-DL outperforms by a large margin the traditional solutions based on ImageNet pre-training (respectively, by 26% and 14% for the two tasks with respect to ImageNet-collapsed solution, and by 11% for the first task with respect to ImageNet-concatenated solution). Finally, our GAN-based approach outperforms by a good margin the other SSRL method based on convolutional autoencoder, especially in the $C^+$ versus $C^-$ task.

The last two lines of Table 1 report the accuracy of the $C^+$ versus $C^-$ categorization task, this time separated by the cellular models HRCE and VERO. For all the considered embeddings, we can observe that the accuracy is generally higher when the cell models are separated. Nonetheless, this variation is quite contained for the SSRL solutions. More specifically, GAN-DL shows an accuracy of 92.44% and 99.93% for respectively HRCE and VERO, against the 91.4% obtained with the two models considered together. The baseline, on the other hand, shows an accuracy of 99.28% and 100% for respectively HRCE and VERO, against the 95.81% for the two merged cell models. We can again observe that the ImageNet pre-trained solutions reported a much higher accuracy difference: 84.09% and 84.53% against 65.31% for the ImageNet-collapsed solution, and 90.24% and 99.8% against 79.61% for the ImageNet-concatenated strategy. Finally, even in this configuration, the embedding based on a convolutional autoencoder obtained the lowest accuracy values.

**Table 1:** Classification accuracy on the downstream tasks

	GAN-DL	Baseline[28] (%)	ImageNet-collapsed (%)	ImageNet-concatenated (%)	ConvAE (%)
$C^+$ versus $C^-$	91.4	95.81	65.31	79.61	64.50
HRCE versus VERO	100.0	100.0	85.52	100.0	99.80
$C^+$ versus $C^-$ (HRCE only)	92.44	99.28	84.09	90.24	68.41
$C^+$ versus $C^-$ (VERO only)	99.93	100	84.53	99.8	82.89

### Automatically deriving dose-response curves from image data

In this section, we exploit the GAN-DL’s featurization projected onto the *On-perturbation* axis of the ${{\textbf {E}}}^2$ space, defined in section *Visualizing GAN-DL’s representation learning capability*, to automatically derive the dose-response of all the 1672 screened compounds in RxRx19a [25] dataset. Even in this case, the featurization is the one obtained from the image generation pre-text, which did not exploit any task-specific annotation.

**Fig. 4:**
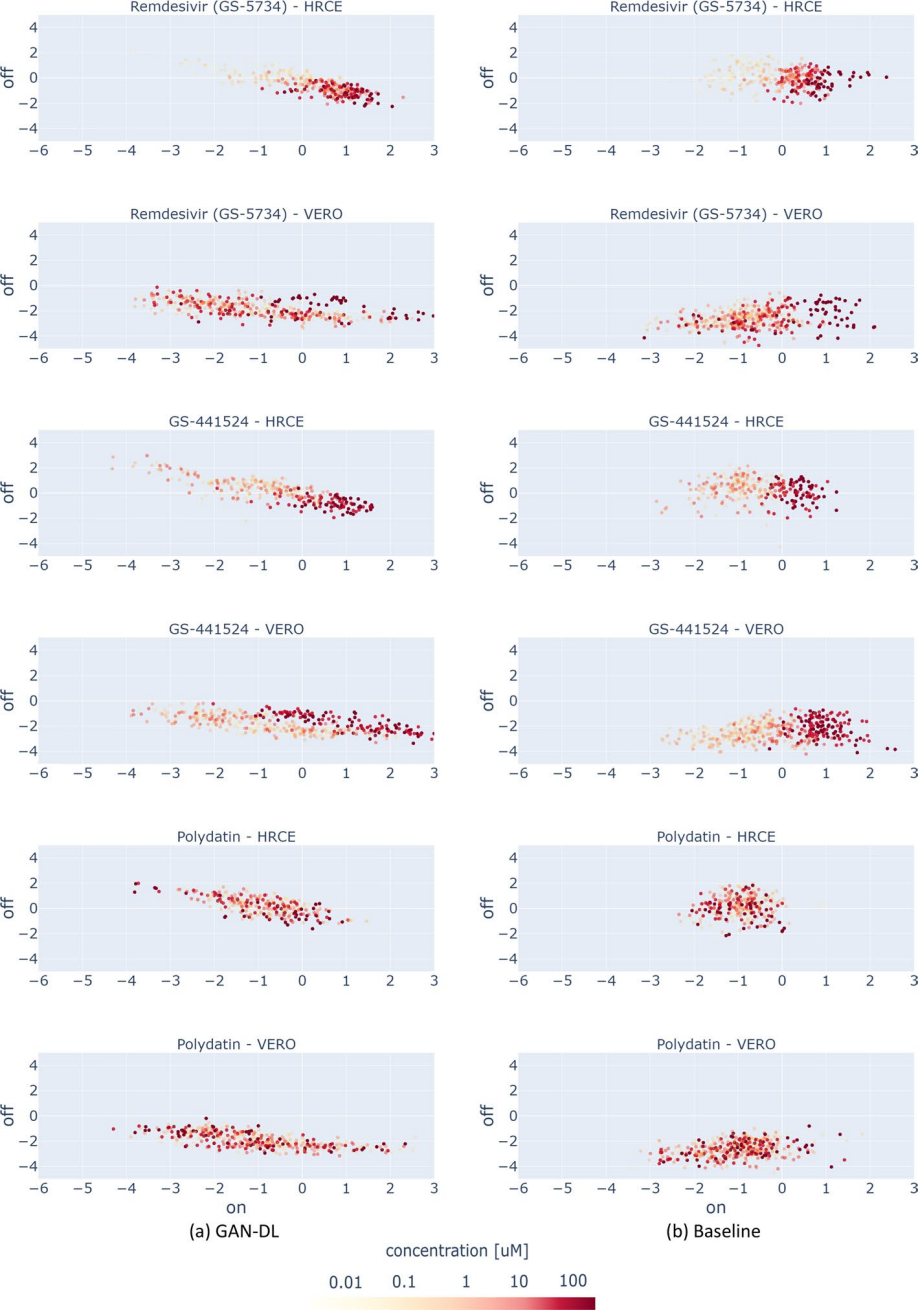
Drug effectiveness as a function of concentration, obtained using our GAN-DL (**a**) and the baseline embedding (**b**)

As the figures of merit we propose: (1) the embedding distributions, in the form of a scatter plot at varying concentrations, of *Remdesivir* and *GS-441524*, two compounds proved to be effective on SARS-CoV-2 in vitro in both the cell models, and of *Polydatin*, a compound that is known to be ineffective [28, 35] (see Fig. 4). These compounds are shown as representative examples for both our embedding (a) and the baseline embedding (b); (2) the dose-response curves of a number of other compounds, obtained by reporting the corresponding mean *efficacy score* at each concentration (see Additional file 1).

From Fig. 4, we can draw a number of considerations. For the effective compounds *Remdesivir* and *GS-441524*, it is possible to see that progressively higher drug concentrations (corresponding to progressively darker red points in the scatter plots) tend to cluster towards positive values of the *On-perturbation* axis in the ${{\textbf {E}}}^2$ space, closer to the region associated to the $C^+$group: the higher the concentration, the higher the *On-perturbation* value. This is generally true for both the GAN-DL and the baseline embedding (see sections (a) and (b) of the figure, respectively), meaning that GAN-DL is equally able to represent the concentration-dependent ability of an active compound to preserve cell viability and inhibit SARS-CoV-2 infection.

Differently from the effective compounds, the ineffective ones should reasonably behave the same in terms of SARS-CoV-2 inactivation, independently of their concentration. When looking at the plot of *Polydatin* (a compound with no known effect on the virus in vitro), the values cluster towards the left side of the on perturbation axis where $C^-$ samples are located and do not show any specific color-pattern at increasing values of dose concentration. This demonstrates that, same as for the baseline, with GAN-DL embedding the ineffective compounds do not show any specific dose-dependent behaviour. Accordingly, very few values of the ineffective compounds are located in the positive *On-perturbation* space (slightly greater then zero), suggesting no inactivation effect for SARS-CoV-2 infection in vitro.

### Zero-shot representation learning

**Fig. 5:**
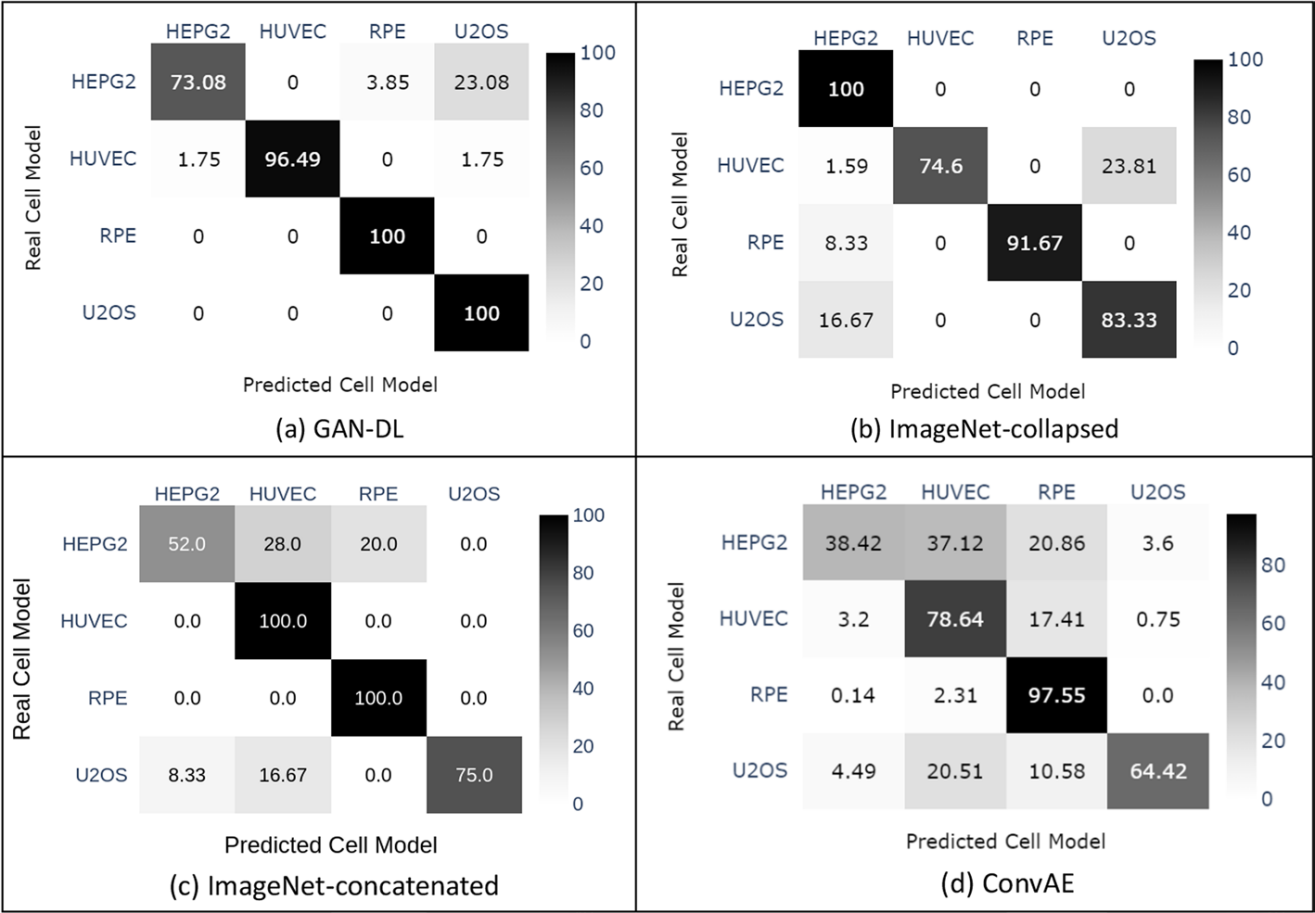
Confusion matrix of the cell classification task on the RxRx1 [26] dataset

In this section we try to assess the generalization capabilities of the proposed embedding technique in a zero-shot representation learning experiment, that consists in a categorization problem where a classifier observes samples described by a featurization learnt not only on a different pretext task, but even on a different dataset. For this purpose, we exploit the RxRx1 [26] image collection, a non-SARS-CoV2 related dataset consisting in 125,510 fluorescent microscopy images featuring human liver cancer cells (HEPG2), human umbilical vein endothelial cells (HUVEC), retinal pigment epithelium cells (RPE) and human bone osteosarcoma epithelial cells (U2OS). For the sake of channels compatibility, to perform a zero-shot inference on the RxRx1 [26] dataset we removed the channel corresponding to the MitoTracker, a dye that stains mitochondria, that is not present in the five-staining protocol of RxRx19a [25]. We exploit a soft margin linear SVM built on top of our GAN-DL embedding to categorize the four different cell models included in the RxRx1 [26] benchmark. We show the corresponding results in the form of a confusion matrix in Fig. 5a. From this matrix we can see that, despite the fact that the RxRx1 [26] cell models are totally new for GAN-DL, they can be linearly separated in the feature space with a mean accuracy of 92.68%.

For comparison, we show the results obtained by: (1) a DenseNet CNN model pre-trained on ImageNet, respectively with collapsed and concatenated strategy; (2) a convolutional autoencoder [4] trained on RxRx19a. As shown in the confusion matrices of Fig. 5, both the DenseNet-based classifiers (ImageNet-collapsed and ImageNet-concatenated) and the convolutional autoencoder (ConvAE) obtained an accuracy at least 5% lower than our GAN-DL.

## Discussion

In contexts where dataset annotation is costly, like medical and computational biology domains, the current standard, for the application of deep learning models on image data, involves the use of a ImageNet-pretrained CNN model [2, 16]. Nevertheless, we found such transfer learning-based strategy totally unsatisfactory for our real word application (see Table 1), where the inherent complexity of the required biological tasks and the experimental set-up of a large scale drug screening initiative claims for a more powerful representation learning technique. If, in general, SSRL seems a promising solution for those scenarios suffering a paucity of labelled data, the recent work by Wallace et al. [4] has shown how traditional SSRL featurization methodologies fail in several biological downstream tasks. This is mainly imputed on the difficulty in defining a pretext task which can be exploited by traditional contrastive SSRL.

On top of these considerations, in this paper we propose GAN-DL, a fully SSRL method leveraging the representation learning acquired by the discriminator of a StyleGAN2 model [19]. Our GAN-DL does not require any task-specific label to obtain the image embedding, as the StyleGAN2 backbone is trained on a generative task based on the competition of a generator and of a discriminator, that is completely independent on the downstream task. By doing so, we address the problem of lack of annotated data, that is instead necessary for conventional CNN-based transfer learning methods. We demonstrated the goodness of our featurization methodology in two downstream supervised tasks: the classification of different cellular models (HRCE vs VERO cells) and the categorization of positive versus negative control groups in the RxRx19a [25] benchmark. For this purpose, we trained a simple linear SVM on top of the self-supervised GAN-DL embedding, which does not require a large number of annotated data. Furthermore, we compared our solution with a baseline state-of-the-art DenseNet model, pre-trained on the RxRx1 dataset [26] (the corresponding embedding is released together with the imaging data by [28]).

On the one hand, the baseline embedding is generally more accurate than GAN-DL in the downstream classification tasks, even though by a small margin. On the other hand, the baseline is pre-trained on a very large annotated dataset (RxRx1 [26] dataset, consisting of 296 GB of fluorescent microscopy images), while training GAN-DL does not require any task-specific image annotations. This is indeed a major advantage for the re-usability of our method in different contexts where annotated data from a similar domain are few or even not available at all, which is a frequent challenge of many biological applications [2, 4].

We furthermore compare our GAN-DL with ImageNet-pretrained models, traditionally exploited as fixed feature extractor for biological images [2, 36, 37], as well as with an other SSRL method based on convolutional autoencoder. We found our GAN-DL superior to both the ImageNet-based strategies and to the convolutional autoencoder, the latter resulting less accurate with respect to ImageNet-based strategies by a narrow margin. We believe that the quality of representations extracted by the convolutional autoencoder approach is less competitive than the ImageNet-based methods due to the limited capability of the autoencoder in generating high-quality images (see some illustrative examples in the Additional file 1). The goodness of the results obtained by our GAN-DL, whose backbone is StyleGAN2 [19], state-of-the-art technology in image generation, corroborates the insight that the SSRL adversarial pretext task of learning to generate high-quality synthesized images allows to extract an SSRL representation featuring inherent relations that are not captured by previous techniques.

We speculate that our GAN-DL embedding, leveraging as pre-text task the generation of plausible and high resolution images through the adversarial game between the generator and the discriminator, proficiently learns an unbiased image featurization able to describe the fine-grained patterns that are typical of biological applications. This leads to an improved capability of separating multiple unrelated features along different axis of the latent space, which should be ultimately helpful to address any downstream tasks requiring knowledge of the salient attributes of the data [38]. To demonstrate our claim, we put this capability of GAN-DL into effect in a number of different applications: (i) the classification of active and inactive compounds against SARS-CoV-2 infection in two different cell lines; (ii) the generation of dose-response curves for the large scale molecule screening of RxRx19a [25], without the need of any training on purposely labelled data; (iii) the zero-shot representation learning of four different cell lines included in the RxRx1 [26] dataset.

In conclusion, the satisfactory results obtained in all the presented scenarios on the one hand demonstrate the goodness and generalization capability of our approach, on the other hand legitimize the future exploitation of generative SSRL even in other biological applications, where the collection of annotated images is typically a cumbersome task.

## Supplementary Information

**Additional file 1.** The file contains additional information on the experimental setup and dose response curves obtained using our technique.

## Data Availability

The data underlying this article are available on Recursion’s website at rxrx.ai The RxRx19a dataset is avaliable at https://www.rxrx.ai/rxrx19a#Download The RxRx1 dataset is available at https://www.rxrx.ai/rxrx1#Download.

## References

[1] Liu X, Zhang F, Hou Z, Wang Z, Mian L, Zhang J, Tang J. Self-supervised learning: generative or contrastive, 2020;1(2). arXiv preprint. arXiv:2006.08218.

[2] Ponzio F, Urgese G, Ficarra E, Di Cataldo S (2019). Dealing with lack of training data for convolutional neural networks: the case of digital pathology. Electronics.

[3] Melinscak M, Loncaric S. Retinal oct image segmentation: how well do algorithms generalize or how transferable are the data? 2020. 10.23919/MIPRO48935.2020.9245336.

[4] Wallace B, Hariharan B. Extending and analyzing self-supervised learning across domains. 2020. arXiv:2004.11992.

[5] Ponzio F, Deodato G, Macii E, Di Cataldo S, Ficarra E. Exploiting “uncertain” deep networks for data cleaning in digital pathology. In: IEEE 17th international symposium on biomedical imaging (ISBI). IEEE; 2020. p. 1139–43.

[6] Zhuang F, Qi Z, Duan K, Xi D, Zhu Y, Zhu H, Xiong H, He Q (2020). A comprehensive survey on transfer learning. Proc IEEE.

[7] Farahani A, Voghoei S, Rasheed K, Arabnia HR (2021). A brief review of domain adaptation. Adv Data Sci Inf Eng.

[8] Sajun AR, Zualkernan I (2022). Survey on implementations of generative adversarial networks for semi-supervised learning. Appl Sci.

[9] Van Engelen JE, Hoos HH (2020). A survey on semi-supervised learning. Mach Learn.

[10] Jing L, Tian Y (2020). Self-supervised visual feature learning with deep neural networks: a survey. IEEE Trans Pattern Anal Mach Intell.

[11] Radford A, Kim JW, Hallacy C, Ramesh A, Goh G, Agarwal S, Sastry G, Askell A, Mishkin P, Clark J. Learning transferable visual models from natural language supervision. In: International conference on machine learning. PMLR; 2021. p. 8748–8763.

[12] AAAI 2020 conference. 2020. https://aaai.org/Conferences/AAAI-20/. Accessed 29 April 2021.

[13] Goldsborough P, Pawlowski N, Caicedo JC, Singh S, Carpenter AE. Cytogan: generative modeling of cell images. BioRxiv 2017; p. 227645.

[14] Gildenblat J, Klaiman E. Self-supervised similarity learning for digital pathology. arXiv preprint. arXiv:1905.08139 (2019)

[15] Abbet C, Zlobec I, Bozorgtabar B, Thiran J-P. Divide-and-rule: self-supervised learning for survival analysis in colorectal cancer. In: International conference on medical image computing and computer-assisted intervention. Springer; 2020. p. 480–89.

[16] Yang P, Hong Z, Yin X, Zhu C, Jiang R. Self-supervised visual representation learning for histopathological images. In: International conference on medical image computing and computer-assisted intervention. Springer; 2021. p. 47–57.

[17] Sahasrabudhe M, Christodoulidis S, Salgado R, Michiels S, Loi S, André F, Paragios N, Vakalopoulou M. Self-supervised nuclei segmentation in histopathological images using attention. In: International conference on medical image computing and computer-assisted intervention. Springer; 2020. p. 393–402.

[18] Dmitrenko, A., Masiero, M.M., Zamboni, N.: Self-supervised learning for analysis of temporal and morphological drug effects in cancer cell imaging data. arXiv preprint. arXiv:2203.04289 (2022).

[19] Karras T, Laine S, Aittala M, Hellsten J, Lehtinen J, Aila T. Analyzing and improving the image quality of stylegan. In: Proceedings of the IEEE/CVF conference on computer vision and pattern recognition; 2020. p. 8110–19.

[20] Radford A, Metz L, Chintala S. Unsupervised representation learning with deep convolutional generative adversarial networks. arXiv:arXiv:1511.06434 (2016).

[21] Lin D, Fu K, Wang Y, Xu G, Sun X (2017). MARTA GANs: unsupervised representation learning for remote sensing image classification. IEEE Geosci Remote Sens Lett.

[22] Zhang M, Gong M, Mao Y, Li J, Wu Y (2018). Unsupervised feature extraction in hyperspectral images based on wasserstein generative adversarial network. IEEE Trans Geosci Remote Sens.

[23] Mao X, Su Z, Tan PS, Chow JK, Wang Y-H. Is discriminator a good feature extractor? arXiv:arXiv:1912.00789 (2020)

[24] Arjovsky M, Chintala S, Bottou L. Wasserstein gan. arXiv:arXiv:1701.07875 (2017).

[25] RecursionAI: RxRx19 dataset. https://www.rxrx.ai/rxrx19 (2020).

[26] RecursionAI. RxRx1 dataset. https://www.rxrx.ai/rxrx1 (2019).

[27] Taylor J, Earnshaw B, Mabey B, Victors M, Yosinski J. Rxrx1: an image set for cellular morphological variation across many experimental batches. In: The 7th international conference on learning representations; 2019.

[28] Cuccarese MF, Earnshaw BA, Heiser K, Fogelson B, Davis CT, McLean PF, Gordon HB, Skelly K-R, Weathersby FL, Rodic V, Quigley IK, Pastuzyn ED, Mendivil BM, Lazar NH, Brooks CA, Carpenter J, Jacobson P, lazier SW, Ford J, Jensen JD, Campbell ND, Statnick MA, Low AS, Thomas KR, Carpenter AE, Hegde SS, Alfa RW, Victors ML, Haque IS, Chong YT, Gibson CC. Functional immune mapping with deep-learning enabled phenomics applied toimmunomodulatory and covid-19 drug discovery. bioRxiv2020. 10.1101/2020.04.21.054387. https://www.biorxiv.org/content/early/2020/08/03/2020.08.02.233064.full.pdf.

[29] Zhuang D, Ibrahim AK (2021). Deep learning for drug discovery: a study of identifying high efficacy drug compounds using a cascade transfer learning approach. Appl Sci.

[30] Saberian MS, Moriarty KP, Olmstead AD, Nabi IR, Jean F, Libbrecht MW, Hamarneh G. Deemd: drug efficacy estimation against SARS-COV-2 based on cell morphology with deep multiple instance learning. arXiv preprint. arXiv:2105.05758 (2021). 10.1109/TMI.2022.3178523 35622798

[31] Heiser K, McLean PF, Davis CT, Fogelson B, Gordon HB, Jacobson P, Hurst B, Miller B, Alfa RW, Earnshaw BA, Victors ML, Chong YT, Haque IS, Low AS, Gibson CC. Identification of potential treatments for covid-19 through artificial intelligence-enabled phenomic analysis of human cells infected with SARS-COV-2. bioRxiv. 10.1101/2020.04.21.054387. https://www.biorxiv.org/content/early/2020/04/23/2020.04.21.054387.full.pdf (2020).

[32] Mescheder L, Geiger A, Nowozin S. Which training methods for GANs do actually converge? arXiv:arXiv1801.04406 (2018).

[33] Deng J, Guo J, Xue N, Zafeiriou S. Arcface: additive angular margin loss for deep face recognition. In: Proceedings of the IEEE/CVF conference on computer vision and pattern recognition, 2019. p. 4690–99.

[34] Pihlgren GG, Sandin F, Liwicki M. Improving image autoencoder embeddings with perceptual loss. arXiv. 10.48550/ARXIV.2001.03444. https://arxiv.org/abs/2001.03444 (2020).

[35] Ko M, Jeon S, Ryu W-S, Kim S (2020). Comparative analysis of antiviral efficacy of FDA-approved drugs against SARS-COV-2 in human lung cells. J Med Virol.

[36] Ponzio F, Macii E, Ficarra E, Di Cataldo S. Colorectal cancer classification using deep convolutional networks. In: Proceedings of the 11th international joint conference on biomedical engineering systems and technologies, vol. 2; 2018. p. 58–66.

[37] Mormont R, Geurts P, Marée R. Comparison of deep transfer learning strategies for digital pathology. In: Proceedings of the IEEE conference on computer vision and pattern recognition workshopsm, 2018. p. 2262–71.

[38] Chen X, Duan Y, Houthooft R, Schulman J, Sutskever I, Abbeel P. Infogan: interpretable representation learning by information maximizing generative adversarial nets. arXiv preprint. arXiv:1606.03657 (2016).

