# Supplementary material for: Exploiting generative self-supervised learning for the assessment of biological images with lack of annotations
Source: BMC Bioinformatics. 2022 Jul 24;23:295. doi: 10.1186/s12859-022-04845-1 (PMC9308954; doi:10.1186/s12859-022-04845-1)
Supplement: Supplementary file 1 — Additional file 1. The file contains additional information on the experimental setup and dose response curves obtained using our technique. [file 12859_2022_4845_MOESM1_ESM.pdf]

RESEARCH

Exploiting generative self-supervised learning for the assessment of biological images with lack of annotations - Supplementary Material

Alessio Mascolini[1*†], Dario Cardamone[2,4†], Francesco Ponzio[1], Santa Di Cataldo[1^] and Elisa Ficarra[3^]

Research supported with Cloud TPUs from Google's TensorFlow Research Cloud (TFRC)

*Correspondence:
alessio.mascolini@polito.it
[1]Polytechnic University of Turin, Corso Duca Degli Abbruzzi, Turin, IT
[2]University of Turin, Via Giuseppe Verdi, Turin, IT
[3]University of Modena e Reggio Emilia, Via Università, Modena, IT
[4]Toscana Life Sciences Foundation, Siena, IT
Full list of author information is available at the end of the article
†Equal contributor ^Equal contributor

While Figure 4 in the main article shows representative examples of compounds whose effectiveness in both cell models is a-priori known [1, 2], in the following we provide the dose-response curves obtained with GAN-DL for all the screening compounds, of either known or unknown effectiveness (see Figure 1 of Supplementary Materials). For both HRCE (a) and VERO (b), this figure shows on the x-axis the concentration values and on the y-axis the *efficacy score* of the different compounds. To obtain the efficacy score axis, we normalize the *On-perturbation* values using the controls, so that the mean of the negative controls is -1 and the mean of the positive controls is 1. By doing so, we obtain that the 0 value represents the *efficacy threshold*, i.e. the value above which a

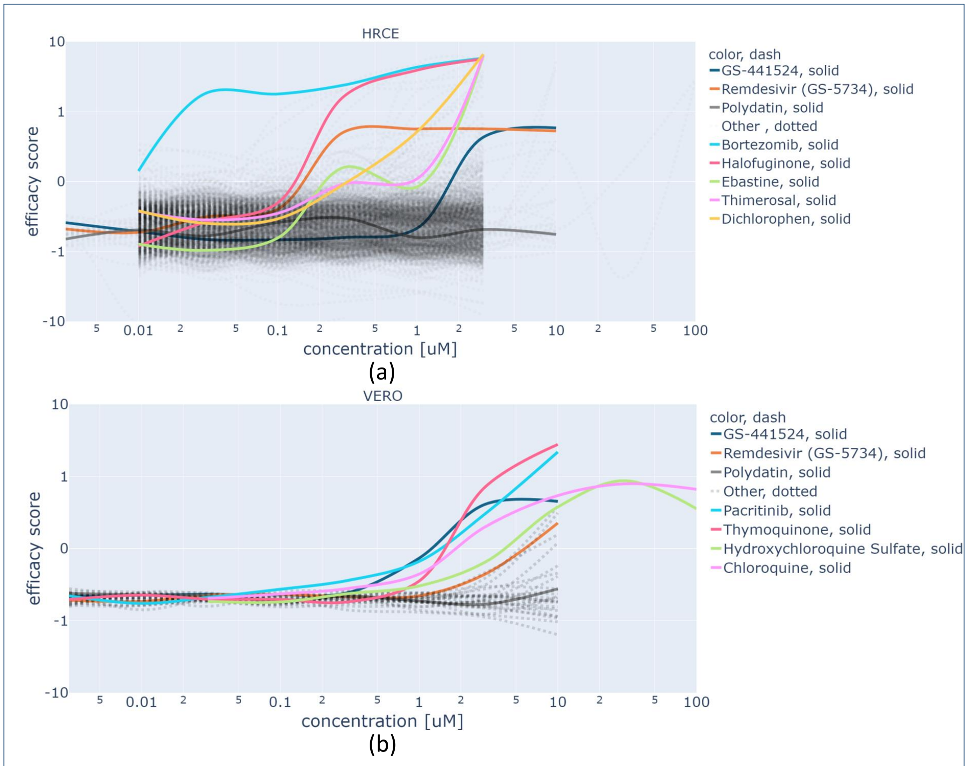

Figure 1: Dose response curves for HRCE (a) and VERO (b) cell models obtained leveraging our GAN-DL's embedding.

compound is considered effective against SARS-CoV-2 infection in vitro. This normalization is performed on each cell model independently.
The curves of the three representative compounds reported in Figure 4 of the main article, are also shown in Figure 1, with solid colored lines to highlight them: *GS-441524* (blue), *Remdesivir* (orange) and *Polydatin* (grey). As it can be gathered from the figure, from a certain concentration value the curves of *GS-441524* and *Remdesivir* are above the efficacy threshold of zero. As the two cellular model behave differently upon SARS-CoV-2 infection, the concentration level above which a compound is effective is specific for the considered cell model. This is an expected typical trend for an effective compound. On the contrary, the *efficacy score* curves of *Polydatin* are always below the value of zero, regardless the tested concentration. This confirms the expected ineffectiveness of the compound. Besides *GS-441524*, *Remdesivir* and *Polydatin*, Figure 1 shows solid colored lines also for the five compounds that obtained the highest efficacy scores in our screening. *Bortezomib, Halofuginone, Ebastine, Thimerosal, Dichlorophen* tested the most effective in HRCE, while *Pacritinib, Thymoquinone, Hydroxychloroquine Sulfate, Chloroquine* in VERO cells. For the sake of readability, all the remaining curves, associated with all the other tested compounds, are reported dashed grey and without a corresponding label.
In general, we can identify three different behaviors: i) under-threshold curves showing no specific correlation between concentration and efficacy score, same as *Polydatin*; ii) almost-monotonically increasing dose response curves, featuring a positive correlation between concentration and efficacy: this is the most expected behavior for an effective compound, where a treatment requires a minimum dose to be efficacious; iii) dose-response curves that are above the efficacy threshold, but start decreasing after achieving a maximum efficacy score at a certain concentration value (see for instance *Hydroxychloroquine Sulfate*, green solid line for the VERO cells). This is the case of a few compounds that were tested at high concentration values (100 uM). Hence, the drop of efficacy score can be reasonably explained by a loss of viability of the cell model related to a toxic effect of the compound at that high concentration.

Experimental setup

GAN-DL

The StyleGAN2 backbone was trained on the RxRx19a [3] dataset using Adam optimizer with a learning rate of 10^{-4}, with the same loss as the one described in the StyleGAN2 paper [4]. No hyperparameter optimization was performed.
Conversely, we employed two regularization terms:

- Generator: Jacobian Regularization (a.k.a Perceptual Path Length regularization) [4], Exponential Moving Average of the weights [5]
- Discriminator: Lipschitz L1 penalty [6], R1 regularization [7]

For training we employed one TPU v3-8 node with 16GiB of RAM per core. TPUs are AI accelerator ASICs (Application Specific Integrated Circuits) which have the ability to train neural networks significantly faster than GPUs by executing a larger amount of computations in parallel.
The original StyleGAN2 took 9 days on 8 Tesla V100 GPUs to train on the FFHQ dataset [4], while our slimmed and repurposed version required 24 hours on a

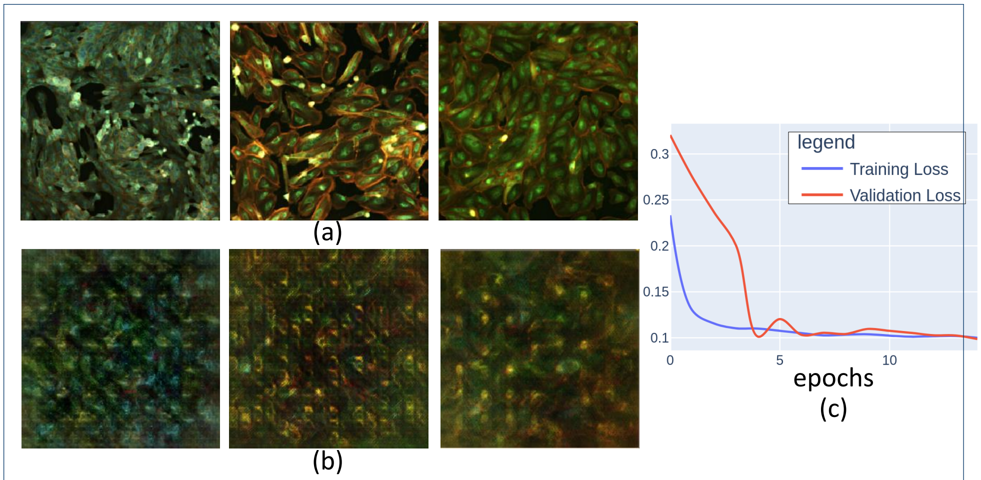

Figure 2: Illustrative examples of RxRx19a [8](a) and the corresponding images reconstructed by the ConvAE approach (b). ConvAE's training and validation curves (c).

TPU v3-8 node or 48 hours on a single Tesla V100 GPU to obtain the results shown in this paper. Most of the difference in training time can be attributed to the lower amount of parameters as well as the vastly different dataset used.

ConvAE

The embedding of the ConvAE was obtained by training the model on the target dataset RxRx19a [8], employing an NVIDIA TITAN Xp GPU. To ensure convergence on the high resolution RxRx19a images, we modified the original autoencoder architecture presented by Wallace et al. [9] in the following way: we employed the same residual connection scheme used in the generator of StyleGAN2 and GAN-DL and a perceptual loss function obtained using an Imagenet pretrained ResNet50 [10]. The learning rate was set to 10^{-4} and the training leverages Adam optimizer. Figure 2 provides some representative examples of ConvAE reconstructed images (b), alongside the original inputs (a), for the RxRx19a [8] dataset. Lastly, Figure 2(c) provides the training and validation trends of the ConvAE's loss with respect to training epochs.

Author details

[1]Polytechnic University of Turin, Corso Duca Degli Abbruzzi, Turin, IT. [2]University of Turin, Via Giuseppe Verdi, Turin, IT. [3]University of Modena e Reggio Emilia, Via Università, Modena, IT. [4]Toscana Life Sciences Foundation, Siena, IT.

References

1. Cuccarese, M.F., Earnshaw, B.A., Heiser, K., Fogelson, B., Davis, C.T., McLean, P.F., Gordon, H.B., Skelly, K.-R., Weathersby, F.L., Rodic, V., Quigley, I.K., Pastuzyn, E.D., Mendivil, B.M., Lazar, N.H., Brooks, C.A., Carpenter, J., Jacobson, P., Glazier, S.W., Ford, J., Jensen, J.D., Campbell, N.D., Statnick, M.A., Low, A.S., Thomas, K.R., Carpenter, A.E., Hegde, S.S., Alfa, R.W., Victors, M.L., Haque, I.S., Chong, Y.T., Gibson, C.C.: Functional immune mapping with deep-learning enabled phenomics applied to immunomodulatory and covid-19 drug discovery. bioRxiv (2020). doi:10.1101/2020.08.02.233064. https://www.biorxiv.org/content/early/2020/08/03/2020.08.02.233064.full.pdf
2. Ko, M., Jeon, S., Ryu, W.-S., Kim, S.: Comparative analysis of antiviral efficacy of fda-approved drugs against sars-cov-2 in human lung cells. Journal of medical virology (2020)
3. RecursionAI: RxRx1 dataset. https://www.rxrx.ai/rxrx1 (2019)
4. Karras, T., Laine, S., Aittala, M., Hellsten, J., Lehtinen, J., Aila, T.: Analyzing and improving the image quality of stylegan. In: Proceedings of the IEEE/CVF Conference on Computer Vision and Pattern Recognition, pp. 8110–8119 (2020)

5. Karras, T., Aila, T., Laine, S., Lehtinen, J.: Progressive growing of gans for improved quality, stability, and variation. arXiv (2018). 1710.10196
6. Petzka, H., Fischer, A., Lukovnicov, D.: On the regularization of wasserstein gans. arXiv (2018). 1709.08894
7. Mescheder, L., Geiger, A., Nowozin, S.: Which training methods for gans do actually converge? arXiv (2018). 1801.04406
8. RecursionAI: RxRx19 dataset. https://www.rxrx.ai/rxrx19 (2020)
9. Wallace, B., Hariharan, B.: Extending and Analyzing Self-Supervised Learning Across Domains (2020). 2004.11992
10. Pihlgren, G.G., Sandin, F., Liwicki, M.: Improving Image Autoencoder Embeddings with Perceptual Loss. arXiv (2020). doi:10.48550/ARXIV.2001.03444. https://arxiv.org/abs/2001.03444
